# Epigenetic Switches in Retinal Homeostasis and Target for Drug Development

**DOI:** 10.3390/ijms25052840

**Published:** 2024-02-29

**Authors:** Kalpana Rajanala, Arun Upadhyay

**Affiliations:** Ocugen Inc., 11 Great Valley Parkway, Malvern, PA 19355, USA; kalpana.rajanala@ocugen.com

**Keywords:** age related macular degeneration, chromatin remodeling, epigenetics, homeostasis, inherited retinal disorders, non-coding RNAs, nuclear hormone receptors, retina development

## Abstract

Retinal homeostasis, a tightly regulated process maintaining the functional integrity of the retina, is vital for visual function. Emerging research has unveiled the critical role of epigenetic regulation in controlling gene expression patterns during retinal development, maintenance, and response to mutational loads and injuries. Epigenetic switches, including DNA methylation, histone modifications, and non-coding RNAs, play pivotal roles in orchestrating retinal gene expression and cellular responses through various intracellular, extracellular, and environmental modulators. This review compiles the current knowledge on epigenetic switches in retinal homeostasis, providing a deeper understanding of their impact on retinal structural integrity and function and using them as potential targets for therapeutic interventions.

## 1. Introduction

The retina is a complex and highly specialized neural tissue in the eye responsible for converting light into electrical signals for visual perception. The retina is comprised of different cell types stratified as distinct layers. The rod and cone photoreceptors constitute the outer nuclear layer (ONL); the synapses of the photoreceptor axons form the outer plexiform layer (OPL); the bipolar, Müller, horizontal, and amacrine cells constitute the inner nuclear layer (INL); the inner plexiform layer (IPL) is made up of the synaptic connections between bipolar cell axons and ganglion cell dendrites; and the retinal ganglion cells form the ganglion cell layer. Photoreceptors, especially, are highly specialized cells of the retina that have enhanced metabolic demands due to high turnover of proteins and continuous exposure to photo-oxidative stress and require constant clearance of reactive oxygen species and retinoid byproducts. To maintain the retina’s function and structure, retinal homeostasis is crucial, involving intricate regulatory mechanisms at the genetic and epigenetic levels.

The term epigenetics describes variations in gene expression patterns brought on by alterations to genomic structure rather than changes to the DNA sequence. Epigenetic regulation influenced by intrinsic and environmental factors results in activation or suppression of target gene expression. Epigenetic modifications in response to intrinsic and external stimuli impart a novel level of regulation in retinal development and maintenance. Studies have identified DNA methylation, histone modifications, chromosomal organization, microRNAs, long non-coding RNAs, and nuclear hormone receptors as key mediators of epigenetic regulation in the retina. Dysregulation of this mechanism has been implicated in a variety of retinal diseases, including retinal fibrosis, diabetic retinopathy, retinoblastoma, retinitis pigmentosa (RP), and age-related macular degeneration (AMD).

It is essential to understand the molecular epigenetic mechanisms underlying the onset and development of retinal degenerative diseases. Advancements in technology that can provide a genome-wide assessment of epigenetic modifications like DNA methylation have imparted a deeper insight into differential epigenetic patterns at various developmental stages and diseases. Recognizing and interpreting epigenetic signatures associated with the etiology and pathogenesis of retinal diseases will assist in the development of novel and effective therapies. This review article focuses on the emerging research related to epigenetic switches and their roles in retinal homeostasis.

## 2. Epigenetic Mechanisms in the Retina

### 2.1. Role of Nuclear Receptors

**Figure 1:**
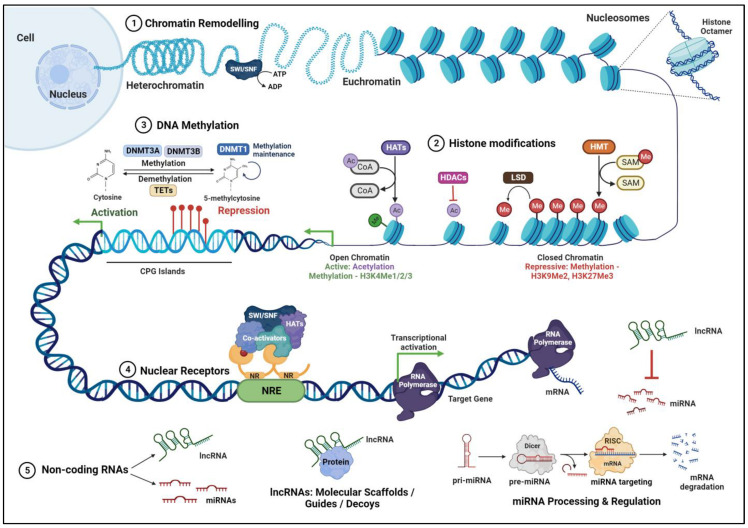
Levels of epigenetic regulation—Chromatin remodeling complexes such as SWI/SNF (switch/sucrose non-fermenting) complexes Histone post translational modifications mediated by histone methyltransferases (HMTs), histone acetyltransferases (HATs), and histone deacetylases (HDACs) DNA methylation catalyzed by DNA methyltransferase enzymes (DNMTs), nuclear receptors (NRs), and noncoding RNAs such as long non-coding RNAs (lncRNAs) and microRNAs (miRNAs) modulate transcriptional activity of the genome. The details of specific processes are elaborated in the text. SAM: S-adenosyl-methionine; LSD: Lysine specific demethylase 1; TET: ten-eleven translocation methyl cytosine dioxygenases; CPG: 5′-CpG-3′ dinucleotides; NRE: Nuclear Receptor Response Element. Symbols: Me: Methylation; Ac: Acetylation; The red circle and lines on CPG islands denote Methylation. The figure was generated using Biorender.com.

Nuclear receptors (NRs) are structurally made up of an amino-terminal domain, followed by a DNA-binding domain (DBD) and the carboxy (C)-terminal ligand-binding domain (LBD). These ligand-dependent transcription factors bind to DNA nuclear receptor response elements (NRRE) on the promoter region of the target genes (Figure 1). The primary function of a nuclear receptor is to regulate target gene expression as an adaptive response to variations in steroid, environmental, and endogenous compounds. NRs include receptors with known ligands such as retinoids or thyroid hormones (RARs and TRβ2) and orphan receptors such as NR2E3, RORα, RORβ, and COUP-TF, which have unspecified functional ligands. The nuclear hormone receptor superfamily of ligand-dependent transcription factors includes NR2E3 (Nuclear receptor subfamily 2 group E member 3), also known as photoreceptor-specific nuclear receptor [1,2]. NR2E3 functions as a transcriptional initiator of many genes specific to rods, controlling the growth of photoreceptor cells [2,3]. Retinoic acid receptor-related orphan receptor (ROR) β acts upstream of the transcription factor NRL and regulates the development of photoreceptor outer segments. RORα plays a significant role in the development of cones, especially by regulating the transcription of S-opsin and M-opsin cone genes by directly binding to the Opn1sw promoter [4]. Human photoreceptors constitute ~70% of the retina, and they maintain their function for a lifetime. Studies have implicated a role for estrogen-related orphan receptor β (ERRβ) in regulating ATP generation and consumption in photoreceptors, contributing to their long-term survival and function [5]. Liver X receptors (LXRs), also known as nuclear receptor subfamily 1 group H (NR1H) receptors, are cholesterol-sensing transcription factors that are known to form heterodimers with RXRs and modulate glucose and cholesterol homeostasis [6].

### 2.2. DNA Methylation

DNA methylation is a process that involves the methyl group transfer from S-adenosyl methionine to cytosine residue in 5′-CpG-3′ dinucleotides (CpG), resulting in a 5-methyl cytosine base (Figure 1). This modification leads to the recruitment of transcriptional repressor complexes to methylated promoter regions, typically resulting in gene silencing. However, depending on the genomic context, it can also be involved in gene activation. DNA methylation is catalyzed by DNA methyltransferase enzymes (DNMTs), comprising the isoforms DNMT1, DNMT2, DNMT3A, DNMT3B, DNMT3C, and DNMT3L. DNMT1 maintains DNA methylation patterns during DNA replication in the newly synthesized daughter DNA strands. DNMT3A and DNMT3B generate de novo DNA methylation patterns in specific differentiated cells and tissues. During embryonic development, the DNA methylation pattern regulates the cell differentiation process, ultimately determining the cell lineage by marking specific genes to be expressed by each cell type [7]. The genomic methylation profile is established by DNMT3 A and B during the developmental process and is maintained during the subsequent cell divisions by DNMT1. Methylated DNA is demethylated through an active or a passive demethylation pathway. Active demethylation is catalyzed by ten-eleven translocation methyl cytosine dioxygenases (TETs), which promote the progressive oxidation of 5-methyl cytosine to produce 5-hydroxymethyl cytosine. The 5-hydroxymethyl cytosine is oxidized further to 5-formylcytosine and 5-carboxylcytosine, which are then modified and excised by thymine DNA glycosylases (TDGs) (Figure 1), subsequently resulting in the replacement of methylated cytosine with unmethylated cytosine. Passive demethylation is referred to as the loss of methylation in the newly synthesized DNA strand during the replication process.

Recent studies have identified key DNA methyltransferases and demethylases in the retina and their impact on retinal development and maintenance. In the retina, the cone and rod specific genes exhibit distinct patterns of DNA methylation, which is required for cell type restricted gene expression. In the retina, DNMT1-mediated DNA methylation was shown to be critical for the expansion of the retinal progenitor pool and for the survival and maturation of postmitotic neurons [8]. Photoreceptor specific genes such as *RHO* (rhodopsin), *RBP3* (retinal binding protein 3), *OPN1SW* (cone opsin, short-wave-sensitive), *OPN1MW* (cone opsin, middle-wave-sensitive), and *OPN1LW* (cone opsin, long-wave-sensitive) were found to be hypomethylated in photoreceptors and methylated in non-photoreceptor cells from the inner nuclear layer suggesting the importance of a differential DNA methylation pattern in modulating photoreceptor gene expression [9].

### 2.3. Histone Modifications

Histones are small basic proteins containing a central globular domain flanked by N- and C-terminal peptides of varying lengths—the histone “tails”. Histone post-translational modifications, such as acetylation, methylation, sumoylation, phosphorylation, and ubiquitination, on core histones H2A, H2B, H3, and H4 regulate chromatin structure and gene accessibility. They play pivotal roles in controlling gene expression during retinal development and adaptation to environmental changes.

Histone acetylation occurs on the lysine residues at the N-terminal end of the core histones H2A, H2B, H3, and H4. The regulation of the acetylated state of histones is carried out by two classes of histone-modifying enzymes: histone acetyltransferases (HATs) and histone deacetylases (HDACs). These enzymes can add or remove acetyl groups to regulate the transcriptional activity and, thereby, the expression of target genes (Figure 1). Mammalian HATs are classified into three main subclasses: The Gnat5 family includes Gcn5, PCAF, Hat1, Elp3, and Hpa2 proteins, which acetylate all core histone proteins; the MYST family, comprising Esa1, MOF, Sas2, Sas3, MORF, Tip60, and Hbo1, which specifically act on the H4 histone; and the p300/CBP family constituting the p300 and CREB-binding protein (CBP), which target H3 and H4 substrates [10]. HATs have specific roles in nucleosome assembly, maintenance of heterochromatin, regulation of transcription, and DNA damage repair [11,12].

Human histone deacetylases (HDACs) that remove the acetyl groups from histones and regulate chromatin structure and transcription are classified into four major classes: class I (HDAC1, HDAC2, HDAC3, and HDAC8); class II, which is further divided into two subclasses: IIa (HDAC4, HDAC5, HDAC7, and HDAC9) and IIb (HDAC6 and HDAC10); and class IV (HDAC11) enzymes, that are zinc ion (Zn^+^) dependent protein deacetylases. Class III deacetylases are NAD^+^ dependent protein enzymes comprising the sirtuin (SIRT) proteins (SIRT1 through 7). SIRT1 was detected in most cellular layers of the retina, including the retinal pigment epithelium (RPE), outer nuclear layer, inner nuclear layer (INL), and ganglion cell layer (GCL) [13]. SIRT1 was shown to protect the retinal cells from inflammation, oxidative stress-induced damage, and apoptotic death [14,15,16]. The absence of a transcription factor for SIRT1, E2f, causes downregulation of the deacetylase activity of SIRT1 and hyperacetylation of p53, resulting in increased apoptosis of the mouse retina [17]. SIRT1 also plays a key role in maintaining energy homeostasis in photoreceptor cells [13]. Interestingly, SIRT1 was shown to interact with DNA methyl transferase DNMT1 and deacetylate the catalytic domain of DNMT1, thereby regulating its activity in cells [18]. SIRT6 regulates glucose homeostasis and maintains retinal function through the deacetylation of the H3K9 and H3K56 sites of multiple genes involved in glycometabolism, such as GLUT1 and GRM6 [19].

Histone methylation is a modification catalyzed by histone methyltransferases (HMTs), which utilize S-adenosylmethionine (SAM) as a substrate to transfer mono-, di-, or trimethyl groups (me1, me2, and me3, respectively) to the basic residues of histones. Histone methylation patterns such as H3K4me3, H3K79me3, and H3K36me3 are typically associated with transcriptional activation, while H3K27me3 and H3K9me3 have been linked to transcriptional silencing [20]. (Figure 1). Mouse tissue array staining indicates that H3K27me3 mainly deposits in ganglion, amacrine, and horizontal cells; however, low levels of H3K27me3 can still be detected in other cell types, including rods [21]. Knockout of G9a (KMT1C), the HMT that acts on histone H3 and methylates H3K9, results in persistent cell proliferation, partial loss of ONL, and severe morphological defects with loss of photoreceptor cells [22]. Lysine specific demethylase 1 (LSD1) regulates gene expression through the demethylation of H3K9me1/2 and H3K4me1/2 [23], and is highly expressed in retinal cells during photoreceptor differentiation. Specific histone-modifying enzymes have been linked to retinal disorders, highlighting their significance in retinal homeostasis. Mutations in the enzymes responsible for histone methylation and demethylation have been implicated in inherited retinal disease and retinitis pigmentosa [24].

### 2.4. Chromatin Remodeling Complexes

Nucleosomes are primary units of chromatin structure comprising cylindrical core histone octamer surrounded by 147 bp of supercoiled DNA. A single 210 kDa nucleosome core particle serves as the basic building block of the chromatin structure, and several units of nucleosomes are arranged as “beads on a string” on an 11 nm chromatin fiber. Further coiling of the fiber creates a solenoid that is 30 nm thick. Subsequent compaction of individual solenoids spatially organizes chromatin fibers. In eukaryotes, chromatin is organized as a highly transcribable euchromatin and a repressed heterochromatin. Condensed chromatin severely restricts the access of specific DNA-binding transcription factors to their binding sites. Chromatin remodeling complexes relax (euchromatin) or condense (heterochromatin) the chromatin and render the DNA sequences accessible to transcription factors, thereby facilitating gene activation or repression. The ATP-dependent chromatin remodeling SWI/SNF (switch/sucrose non-fermenting) complexes (Figure 1) are made up of 10–12 proteins, including the ATPases, Brahma (Brm), and Brm-related gene 1 (Brg1). Brg1 was shown to regulate cell cycle length and survival during retinal development and is critical for modulating retinal size [25]. A subunit of ATP-dependent chromatin remodeling complex, Baf60c was shown to maintain the proliferation of retinal progenitor cells (RPCS) during development via the Notch signaling pathway [26].

### 2.5. Non-Coding RNAs

Non-coding RNAs include microRNAs, long non-coding RNAs, and circular RNAs, which have emerged as key players in post-transcriptional gene regulation. They fine-tune gene expression by binding to target mRNAs, affecting translation and stability. Accumulating evidence indicates that non-coding RNAs are essential for retinal development, cell fate determination, and response to injury [27,28].

#### 2.5.1. MicroRNAs

Mammalian microRNAs (miRNAs) are small regulatory RNAs that are 20–22 bases in length that bind to complementary sequences in mRNAs, typically to the 3′-untranslated regions (3′-UTRs), and regulate over 60% of the human gene expression [29]. Primary miRNAs (pri-miRNAs) are longer RNA precursors of miRNAs that are transcribed by RNA polymerase II as double-stranded hairpin-like structures from DNA sequences and are modified post-transcriptionally by capping, polyadenylation, and splicing [30]. These precursors are cleaved by RNase III endonuclease, the Drosha ribonuclease, and DiGeorge critical region 8 (DGCR8) complexes to form 60–70 bases long intermediates known as pre-miRNA [31,32]. Further processing of the pre-miRNAs is carried out by a second RNase III endonuclease, Dicer, which cleaves the loop of the hairpin, yielding an miRNA duplex: miRNA and miRNA* [33]. RNA-Induced Silencing Complex (RISC) releases the miRNA* strand and rapidly degrades it, yielding mature miRNAs [33]. The binding of mature miRNAs to their target mRNAs results in the silencing of the gene, either by cleavage of mRNA at the binding site, destabilization of the mRNA due to the shortening of the poly(A) tail, or blocking the translation of mRNA [34].

**Figure 2:**
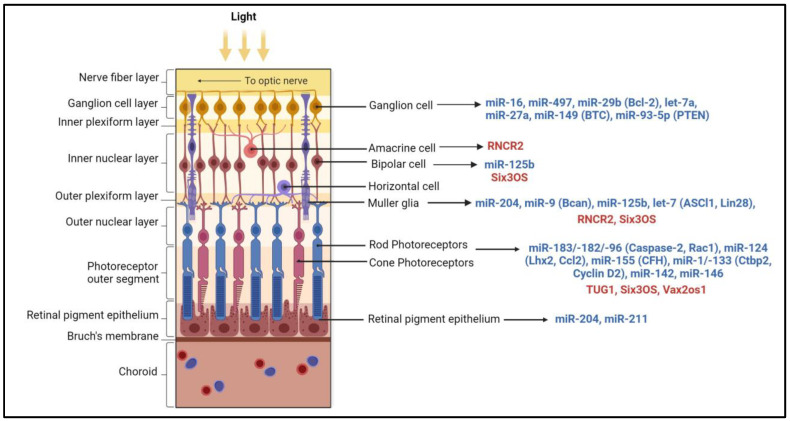
Non-coding RNAs associated with different retinal cell types. The miRNAs (in blue) with increased expression in various retinal cell types and their validated targets are shown in parentheses. The lncRNAs (in red) such as Six3 Opposite Strand (Six3OS), Taurine Upregulated Gene 1 (TUG1), retinal non-coding RNA4 (RNCR4), and Vax2os (ventral anterior homeobox 2, opposite strand) highly expressed in each retinal cell type are indicated. Bcl-2: B-cell lymphoma 2; BTC: betacellulin; PTEN: phosphatase and tensin homologue; Rac: Rac (the Ras-related C3 botulinum toxin substrate); Bcan: brevican proteoglycan; ASCL1: achaete-scute homolog 1; Lhx2: LIM homeobox protein 2; CCL2: C-C Motif Chemokine Ligand 2; CFH: complement factor H; Ctbp2: C-terminal-binding protein 2. The figure was generated using Biorender.com.

Numerous miRNAs were reported to express at various stages of retinal development. In mice, miR-17, miR-18, miR-19, miR-20, miR-93, miR-106, and miR-130 are downregulated, whereas the let-7 family, miR-7, miR-9, miR-9*, miR-96, miR-101, miR-124, miR-181, miR-182, and miR-183 are highly expressed during retinal development [35]. A highly conserved miRNA cluster of miR-183/-182/-96, which is expressed as a single polycistronic transcript and exhibits significant target sequence similarity, regulates microphthalmia-associated transcription factor, which is required for RPE cell differentiation [36,37,38]. This cluster is enriched in rod and cone photoreceptors and is involved in retinal development, including maintenance of cone photoreceptor outer segments, axonal growth, synaptogenesis, and formation of tight conjunctions between Müller glial cells and photoreceptors [39,40,41] (Figure 2). In the mammalian retinas, the miR-183 cluster, miR-204, and miR-211 transcripts are regulated by light, and their total levels are reduced during dark adaptation and upregulated in light conditions [42]. Müller glial cells nourish and protect retinal neurons, support structural integrity, and maintain the homeostasis of the retina [43]. MiRNA-125, miR-9, and let-7 were shown to be critical for the maturation and function of Müller glial cells [44] (Figure 2).

#### 2.5.2. Long Non-Coding RNAs (LncRNAs)

LncRNAs are regulatory non-coding RNA transcripts that are over 200 nt in length. They are transcribed by RNA polymerase II and are post-transcriptionally modified by splicing, capping, and addition of poly-A tails. They can originate from introns, exons, or the intergenic genomic regions and can be transcribed either from the sense or antisense strand of a gene. LncRNAs function in numerous cellular processes, including transcription, translation, splicing, cell growth and differentiation, and regulation of chromatin structure and modifications. They can also act independently at the post-transcriptional level by sponging miRNAs (Figure 1). Dysregulation of LncRNAs alters the proliferation, differentiation, and apoptosis of cells, resulting in pathological changes. LncRNAs associate with chromatin modifiers, such as PRC2 and H3K9 methyltransferases, at specific genomic loci to modulate gene expression. Among the first LncRNAs studied in the retina, Taurine Upregulated Gene 1 (TUG1) was shown to be involved in photoreceptor development and survival [45]. Vax2os1 regulates cell cycle proliferation and progression of photoreceptor progenitors [46,47]. MALAT1, which is expressed in all the layers of the retina, was shown to be critical for the survival of rod photoreceptors [48,49]. Retinal non-coding RNA4 (RNCR4 or BB283400) was shown to modulate pri-miR-183/-182/-96 processing and maintain the proper thickness of photoreceptors and INL of the retina [41]. Various lncRNAs expressed in specific retinal layers are indicated in Figure 2.

#### 2.5.3. Circular RNAs (circRNAs)

Circular RNAs are a sub-type of non-coding RNAs that exhibit evolutionary conservation and tissue-specific expression. A recent study done in mice identified four circRNAs—circHipk2, Cdr1as, circAnkib1, and circTulp4, which had elevated expression levels during retinal development [50]. Circ-Tulp4 was shown to act as miR-26a/671/204 sponge and results in downregulation of the targets such as *Meis2*, *Cdh2*, *Mitf*, and *Pde4b*, which are critical for transcriptional regulation during retinal development [50].

## 3. Epigenetic Regulation during Retinal Development

During retinal development, the precise spatiotemporal regulation of gene expression is essential for the differentiation and maturation of retinal cell types. Epigenetic switches are critical determinants of these processes, dictating cell fate decisions and establishing cell-specific gene expression patterns and maintenance of functions throughout life. Dysregulation and imbalances in these switches can lead to various pathological conditions. Compartmentalization and organization of chromatin in the nucleus play a critical role in retinal development. In the conventional nucleus and the nucleus of the retinal neurons, the euchromatin is centrally localized, and the heterochromatin is adjacent to the nuclear lamina. However, in rod cells, the nuclear organization is inverted, and the heterochromatin is located at the center [51]. The central region of heterochromatin surrounded by euchromatin in rods affects light scattering patterns and optical properties of rod receptors and aids in adaptation to night vision [51].

**Figure 3:**
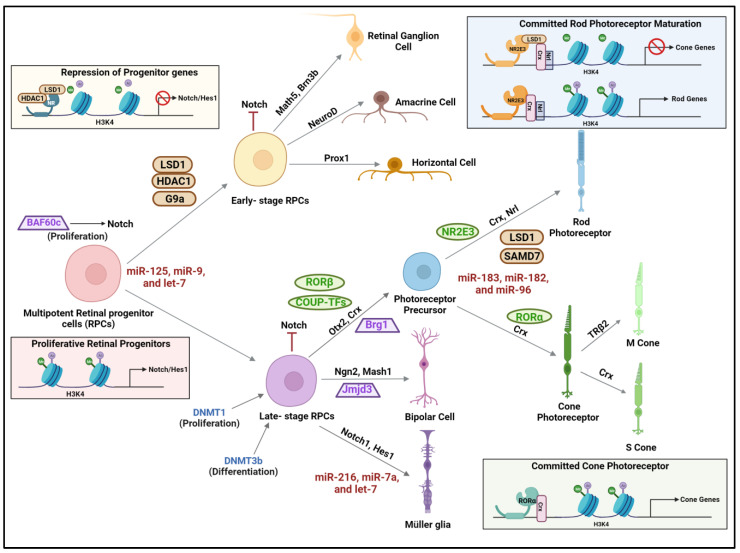
Epigenetic regulation during retinal development—Baf60c maintains the retinal progenitor cells (RPCs) in a proliferative state during development of the retina due to the activation of Notch signaling. The pluripotent genes are silenced during the differentiation of RPCs by histone deacetylation mediated by LSD1 and histone deacetylase 1 (HDAC1) and the repressive methylation of H3K9me2 by histone methyltransferase G9a. Dnmt1 facilitates the proliferation of late-stage retinal progenitor cells. HDAC1 facilitates the differentiation of RPCs by downregulating the multipotent genes. Various miRNAs involved in different stages of development are indicated in red. The nuclear hormone receptors that play specific roles during retinal development are indicated in green. LSD: lysine specific demethylase 1; BRG1: Brahma-related gene 1; Baf60c: BRG1/Brahma -associated factor; JMJD3: Jumonji domain-containing protein D3; Nrl: Neural retina leucine zipper; Brn3b: brain-specific homeobox/POU domain protein 3B; PROX1: prospero homeobox 1; COUP-TFs: chicken ovalbumin upstream promoter transcription factors; HES1: hairy and enhancer of split-1; OTX2: orthodenticle homeobox 2; SMAD7: Small Body Size (SMA) and Mothers Against Decapentaplegic family 7; TRβ2: thyroid hormone receptor β2; NR2E3: Nuclear Receptor Subfamily 2 Group E Member 3. The figure was generated using Biorender.com.

Primitive neuroectoderm located in the anterior part of the neural plate called the retinal field or prospective retina is established by neural patterning coordinated through retinoic acid, Wnt, FGF, TGF-β, and BMP (bone morphogenetic protein) signaling pathways [52,53]. Subsequently, events coordinated through TGF-b (nodal), sonic hedgehog (Shh) signaling, and additional patterning lead to the formation of a pair of optic vesicles, which include optic stalk progenitors and retinal progenitor cells [53]. Multipotent retinal progenitor cells (RPCs) are precursors that differentiate seven different retinal cell types in two stages of histogenesis. During early histogenesis, retinal ganglion cells (RGCs) are the first cells that are generated, followed by the genesis of cone photoreceptors, horizontal cells, and amacrine cells. Bipolar cells, Müller glia, and rod photoreceptors are generated postnatally during late histogenesis [54]. Retinoic acid, vitamin A, and RARβ are important factors for the development and maintenance of the retinal components [55,56]. Key transcription factors such as OTX2 (orthodenticle homeobox 2 protein), CRX (cone-rod homeobox protein), NRL (neural retina leucine zipper protein), TRβ2 (thyroid hormone receptor β2), and nuclear receptors NR2E3 and RORβ regulate the lineage commitment of the retinal progenitors to rod or cone photoreceptors. The rod or cone fate specification is determined by NRL and its downstream target NR2E3. The S or M cone identity is established by TRβ2, and CRX regulates rod and cone receptor maturation [57]. Several molecular studies have implicated *Crx* and *Nrl* genes to be essential for photoreceptor development, and their activity is modulated by HDACs [58] (Figure 3). Ezh2, a central component of polycomb repressive complex 2 (PRC2), catalyzes H3 at lysine 27 (H3K27) methylation resulting in epigenetic silencing [59]. The basic helix-loop-helix family of transcription factors, such as Hes1, Hes5, Hes2, Mash1, Math3, Math5, Neurod1, and Neurod4, act either as activators and repressors to regulate neurogenesis and gliogenesis in the retina. Math5 and Pax6 coordinate the differentiation of retinal progenitors into retinal ganglion cells [60]. The part of the chromatin remodeling complex, Brg1, was shown to regulate genes involved in cell polarity and adhesion and is critical for photoreceptor differentiation and retinal lamination during retinogenesis [25].

The cell-specific methylation pattern in retinal neurons is an indication of active methylation and demethylation processes during retinal development. The rod and the cone cells have varied DNA methylation patterns, and the rod receptors exhibit hypomethylated DNA and a closed chromatin state compared to cones [61]. DNA demethylation is also essential for the development of bipolar cells and the differentiation of photoreceptors. Dnmt1-dependent methylation is especially vital for the retinal progenitor pool expansion and for the maturation and survival of postmitotic neurons [62]. DNMT3B has an essential role in the differentiation of RPCs into precursors of bipolar, horizontal, and photoreceptor cells [53]. DNA methylation marks result in the recruitment of histone H3K9 methyltransferases (HMTs), which then modify the histone tails to help establish a repressed state. In this context, the association between Dnmt3 and an H3K9 methyltransferase, G9a, plays a vital role in the development and differentiation of the eye [63]. In the mouse embryonic lens, HATs CBP and p300 are widely expressed and were shown to catalyze histone H3K9 acetylation [64]. These HATs form complexes with transcription factors such as CRX and NRL to mediate the differentiation of RPCs to photoreceptor cells. In addition, HATs CBP and p300 also interact with transcription factors, such as Pax6, c-Maf, and CREB, to regulate the lens-specific expression of αA-crystallin during lens development [64].

Another level of regulation during retinal development is mediated by non-coding RNAs. MicroRNAs such as let-7, miR-125, and miR-9 are key modulators of the early to late developmental transition of retinal progenitors [44,65] (Figure 3). Let-7 targets cell cycle proteins such as cyclins and cyclin-dependent kinases (CDKs) and control cell cycle progression and exit [66]. Let-7 mi-RNA is significantly upregulated during retinal development, and its expression was correlated with increased transcript levels of *Rhodopsin*, *mGluR6*, and *Glast* transcripts, thereby regulating the differentiation of retinal progenitor cells to late cell types such as amacrine cells, rod photoreceptors, Müller glia, and bipolar cells [67]. The neuronal versus glial fate specification and the terminal differentiation of Müller glia are modulated by miR-216, miR-7a, and let-7 [65]. Among the most abundantly expressed miRNA in the retina, the miRNA cluster of miR-183/-182/-96 regulates signaling pathways involved in photoreceptor differentiation and maintenance [68]. Specifically, the miR-183 cluster targets a paired-box transcription factor, PAX6, that is critical for morphogenesis of the eye [68]. Another abundant microRNA expressed in the vertebrate central nervous system is miR-124 [69]. During retinal development, partial loss of miR-124 resulted in diminished opsin expression and caused photoreceptor death [69]. MiR-9 directly targets components of the Notch signaling pathway, and the cross-regulation between Hes1 and miR-9 was shown to be associated with the progression of retinal differentiation [70]. In addition to miRNAs, lncRNAs also play key roles in modulating various stages of retinal development. In mouse RGCs, lncRNAs such as linc-3a and linc-3b regulate the expression of *Math5*, *Isl1*, and *Pou4f2* genes, which are key modulators of RGC differentiation and patterning. LncRNAs such as Miat, Six3os1, Tug1, Rncr4, Malat-1, and Vax2os determine retinal cell fate during development.

## 4. Epigenetic Changes in Retinal Diseases

### 4.1. Age-Related Macular Degeneration (AMD)

**Figure 4:**
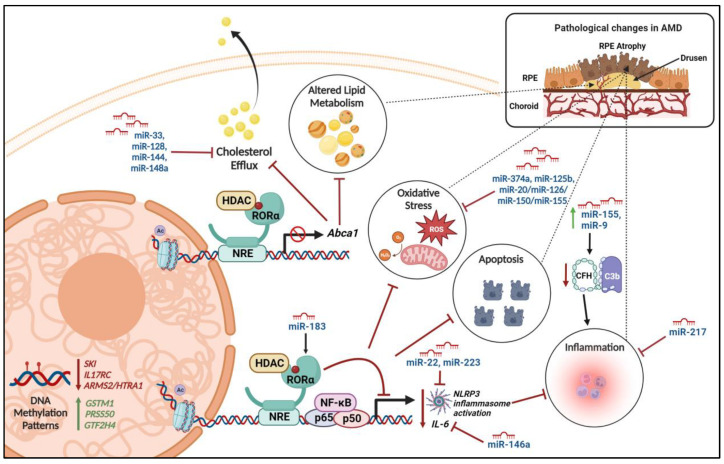
Epigenetic regulation of molecular pathways contributing to AMD—Various epigenetic factors regulate the methylation patterns or alter the transcription levels of genes that are involved in the cellular mechanisms contributing to AMD pathogenesis. IL17RC: interleukin 17 receptor C; CFH: Complement Factor H; ARMS2/HTRA-1: age-related maculopathy susceptibility 2/high-temperature requirement A serine peptidase 1; ROS: reactive oxygen species; NLRP3: NOD-like receptor protein 3; IL6: interleukin-6; NF-κB: nuclear factor-κB; GSTM1: Glutathione S-Transferase Mu 1; PRSS50: protease serine 50; GTF2H4: General Transcription Factor IIH Subunit 4; Abca1: ATP-binding cassette transporter. The figure was generated using Biorender.com.

AMD is a condition causing irreversible vision loss in the elderly. Epigenetic alterations have been implicated in AMD pathogenesis, affecting the expression of genes associated with oxidative stress, inflammation, and angiogenesis. In AMD, several internal and extrinsic stressors lead to the accumulation of extracellular deposits called drusen between the basal lamina of RPE and the inner collagenous layer of Bruch’s membrane. Clusterin/apolipoprotein J is one of the major proteins found in drusen deposits. DNA methylation in the RPE regulates the protein expression of clusterin/apolipoprotein J and inhibits angiogenesis and inflammation. Inhibition of HDACs in the RPE was shown to induce a significant increase in clusterin protein expression and secretion. During the aging process, changes in DNA methylation and histone acetylation status in the cells affect clusterin/apolipoprotein J expression in the RPE and thereby contribute to AMD pathogenesis [71]. DNA methylation is also associated with the antioxidation function in the retina. Oxidative stress response master regulator nuclear factor (erythroid-derived 2)-like 2 (Nrf2) expression is regulated by DNA methylation. Glutathione S-transferases (GSTs) are scavengers of ROS in the cells. In patients with AMD, the higher methylation levels of the glutathione S-transferase promoter cause epigenetic repression of glutathione S-transferase subtypes GSTM1 and GSTM5 in the RPE/choroid and neurosensory retina, making the cells susceptible to oxidative stress [72] (Figure 4). In AMD patients, downregulation of methylation of the interleukin 17 receptor C (IL17RC) promoter that regulates the expression of the IL-17 receptor and modulates the inflammatory responses of IL-17A and IL-17F was reported [73]. Genome-wide methylation analysis of RPE in individuals with AMD revealed differential methylation patterns of multiple genes, including *SKI*, *GTF2H4*, and *TNXB* [74]. SKI protein encoded by the *SKI* gene negatively regulates TGF-β signaling. Reduced SKI expression in the RPE results in increased TGF-β signaling, thereby causing oxidative stress-induced RPE senescence and over-activation of the complement pathway, ultimately contributing to AMD pathogenesis. GTF2H4 is a central component of the highly conserved transcription factor II H (TFIIH) involved in the transcription-dependent nucleotide excision repair of DNA. SIRT1, a class of HDAC, inhibits the activation of an NF-κB-mediated inflammatory pathway. SIRT1 expression was reported to be significantly decreased with increasing age in retinal stem cells, and it was downregulated in human AMD retinas. Dysregulation of miRNA-124 expression and cellular localization results in neuroinflammation and retinal degeneration and is associated with AMD [75]. Particularly, miR-466 and miR-1187 shared common differentially expressed target genes with NRs such as Nr2e3 and RORα that were known to be associated with AMD [76]. LncRNAs have been shown to regulate mitochondrial oxidative stress responses. Prader–Willi region non-protein coding RNA 2 (PWRN2) is upregulated in human ARPE-19 cells upon exposure to oxidative stressors such as H_2_O_2_, tert-butyl hydroperoxide, or UVB [77]. Downregulation of PWRN2 protected human retinal pigment epithelial cells from mitochondrial injuries and apoptosis, implicating PWRN2 to be actively associated with AMD [77]. LINC00167 curbs the expression of miR-203a-3p and increases the expression of the target suppressor of cytokine signaling 3 (SOCS3). LINC00167 causes RPE differentiation, and it was found to be downregulated in patients with AMD [78]. One circular RNA, circZBTB44, which suppresses the expression of miR-578 and regulates endothelial cell proliferation and migration, has been implicated in the pathogenesis of neovascular AMD [79].

Nuclear receptor RORα regulates lipid and cholesterol metabolism [80] and pathways pertaining to lipoproteins, such as serum amyloid A and apolipoprotein A1 [81]. RORα was shown to regulate the expression of SIRT1 and, subsequently, affects deacetylation and nuclear translocation of NF-κB p65 and thereby inhibits the inflammatory response [82] (Figure 4). Ectopic expression of RORα modulates the inflammatory response by interfering with the NF-κB pathway and inhibits TNFα induced expression of IL-6, IL-8, and COX-2 [83]. This anti-inflammatory response and regulation of lipid homeostasis by RORα is mediated by direct interaction with the HDAC corepressor and subsequent suppression of target gene transcription (Figure 4) [84]. RORα SNPs rs4335725 and rs12900948 were shown to be associated with AMD [85]. The miR-183 cluster contains several binding sites for transcription factors such as RORα, indicating a potential for its transcriptional regulation [36]. Upregulated miRNA-9, miRNA-125b, miRNA146a, and miRNA-155 in the retina are associated with complement factor (CFH) deficiency, resulting in inflammatory degeneration in AMD [86]. Especially, miR-146a represses the expression of *IL-6* and *VEGF-A* genes and inactivates the NF-κB signaling pathway in the RPE, which are key factors in the pathogenesis of AMD [87].

### 4.2. Retinal Neurodegenerative Diseases

Inherited retinal degenerative diseases, like retinitis pigmentosa, are characterized by the progressive deterioration of photoreceptor cells. Epigenetic alterations, such as DNA methylation and histone modifications, have been linked to the regulation of genes involved in photoreceptor survival and apoptosis. These modifications were shown to regulate neuronal degeneration in mouse models of retinitis pigmentosa (RP). *Rd1* mice (most used retinitis pigmentosa animal model) retinas exhibit DNA hypermethylation [88], and the inhibition of DNMTs in rd1 retinal explants was shown to reduce photoreceptor cell death [89]. Excess activity of histone deacetylase (HDAC) and poly-ADP-ribose-polymerase (PARP) that are responsible for acetylation and poly-ADP-ribosylation of histones were shown to cause retinal degeneration in *rd1* mice [90,91]. Inhibition of HDAC by Trichostatin A (TSA) resulted in the long-term preservation of cone photoreceptors in *rd1* mice via the regulation of MAPK, PI3K-Akt survival pathways [92]. In an independent study, overexpression of histone deacetylase-4 (HDAC4, a class II HDAC) in *rd1* mice retinas was shown to prolong the survival of rod photoreceptors and bipolar (BP) interneurons by preventing apoptosis [93]. Reduced expression of Sirt1 was found in the mouse retinal degeneration model, which was associated with decreased double DNA stranded DNA-break repair [13]. These results suggest that more than one pathway of photoreceptor survival may be regulated by different classes of HDACs. Inhibition of DNMTs decreases methylation at the Ras-related C3 botulinum toxin substrate 1 (Rac1) promoter, enhances Rac1 activation, and decreases ROS levels in mouse retinal microvessels [94]. Nuclear hormone receptor NR2E3 can modulate gene expression levels associated with RP through epigenetic regulation, and the molecular reset mediated by NR2E3 was shown to reduce retinal degeneration in RP mouse models [95]. A missense variant of NR2E3, c.166G>A, p.(Gly56Arg) or G56R, was reported in 1–2% of retinitis pigmentosa cases [96]. In mouse models of RP, miR-96, miR-182, and miR-183 were downregulated, and miR-1, miR-133, and miR-142 were upregulated [97].

## 5. Epigenetic Therapies for Retinal Disorders

The elucidation of epigenetic mechanisms in retinal homeostasis has created avenues for potential therapeutic interventions. Targeting specific epigenetic regulators may provide novel treatment strategies for retinal diseases, promoting tissue repair and regeneration. An intravitreal injection of the HDAC inhibitor Trichostatin A (TSA) in mice at the late stages of RP was shown to delay cone cell death and prolong their survival [92]. This study provides a lead for targeted pharmacological inhibition of HDAC for the protection of degenerating cones in advanced RP patients to maintain their vision. Tubastatin A mediated inhibition of HDAC6 was shown to protect photoreceptors from ROS by increasing the expression of HSP25, HSP70, and peroxiredoxin 1 [98]. Preclinical studies with HDAC inhibitors provide evidence that they might have neuroprotective effects by reducing TNF-α mediated inflammation and inhibiting apoptosis [99]. 5-Aza-2 0-deoxycytidine (AZA), a DNMT inhibitor, upregulates clusterin expression in RPE cells and inhibits angiogenesis. Hence, AZA can be used as a drug for the prevention of neovascularization in late AMD [71]. HDAC inhibitors such as TSA and valproic acid also induced clusterin expression, thereby inhibiting complement factor mediated inflammation, and, therefore, they can be explored as candidate drugs for AMD.

Gene therapy holds the potential for treating inherited diseases of the eye due to the presence of a blood-retina barrier and allows for the introduction of a foreign antigen to the eye without prompting an aggressive systemic immune response [100]. Expression of nuclear receptor Nr2e3 via AAV8-Nr2e3 gene therapy in multiple unique mouse models of retinitis pigmentosa (RP) and Leber Congenital Amaurosis (LCA) resulted in restoration of photoreceptor cells and improved retinal function [95]. Nuclear hormone receptor RORα, which is involved in the suppression of inflammatory cytokine expression and inhibition of complement factor activation, can be developed as a potential therapy for AMD [101,102]. A recent study demonstrated that gene therapy that delivers RORα decreased drusen-like deposition in Abca4−/− mouse retinas and improved retinal function [102]. The anti-inflammatory properties of microRNA-124 also indicate its utility in therapeutic targeting of retinal diseases such as AMD [75]. A decrease in lncRNA PWRN2 was shown to protect against mitochondrial damage and RPE cell death, and thus targeting this lncRNA can be a therapeutic approach for AMD [77]. Subretinal delivery of AAV-miR-204 increased photoreceptor survival and protected retinal function in the mouse model of dominant retinitis pigmentosa, and in the mouse model of LCA [103].

## 6. Conclusions

Epigenetic switches play fundamental roles in retinal homeostasis, influencing gene expression patterns and cellular responses critical for visual function. Understanding the complex interplay of these epigenetic mechanisms can be highly beneficial for the development of innovative therapies for retinal diseases and potentially enhance visual outcomes for affected individuals. Techniques such as whole-genome methylation sequencing may be helpful in delineating novel biomarkers for the prediction and diagnosis of retinal diseases. Continued research in this area will deepen our knowledge of retinal biology and expand fresh possibilities for future therapeutic interventions.

## References

[1] Kobayashi M., Takezawa S., Hara K., Yu R.T., Umesono Y., Agata K., Taniwaki M., Yasuda K., Umesono K. (1999). Identification of a photoreceptor cell-specific nuclear receptor. Proc. Natl. Acad. Sci. USA.

[2] Haider N.B., Jacobson S.G., Cideciyan A.V., Swiderski R., Streb L.M., Searby C., Beck G., Hockey R., Hanna D.B., Gorman S. (2000). Mutation of a nuclear receptor gene, NR2E3, causes enhanced S cone syndrome, a disorder of retinal cell fate. Nat. Genet..

[3] Cheng H., Khanna H., Oh E.C., Hicks D., Mitton K.P., Swaroop A. (2004). Photoreceptor-specific nuclear receptor NR2E3 functions as a transcriptional activator in rod photoreceptors. Hum. Mol. Genet..

[4] Fujieda H., Bremner R., Mears A.J., Sasaki H. (2009). Retinoic acid receptor-related orphan receptor alpha regulates a subset of cone genes during mouse retinal development. J. Neurochem..

[5] Onishi A., Peng G.H., Poth E.M., Lee D.A., Chen J., Alexis U., de Melo J., Chen S., Blackshaw S. (2010). The orphan nuclear hormone receptor ERRbeta controls rod photoreceptor survival. Proc. Natl. Acad. Sci. USA.

[6] Zelcer N., Tontonoz P. (2006). Liver X receptors as integrators of metabolic and inflammatory signaling. J. Clin. Investig..

[7] Senner C.E. (2011). The role of DNA methylation in mammalian development. Reprod. Biomed. Online.

[8] Nasonkin I.O., Merbs S.L., Lazo K., Oliver V.F., Brooks M., Patel K., Enke R.A., Nellissery J., Jamrich M., Le Y.Z. (2013). Conditional knockdown of DNA methyltransferase 1 reveals a key role of retinal pigment epithelium integrity in photoreceptor outer segment morphogenesis. Development.

[9] Merbs S.L., Khan M.A., Hackler L., Oliver V.F., Wan J., Qian J., Zack D.J. (2012). Cell-specific DNA methylation patterns of retina-specific genes. PLoS ONE.

[10] Roth S.Y., Denu J.M., Allis C.D. (2001). Histone acetyltransferases. Annu. Rev. Biochem..

[11] Grunstein M. (1997). Histone acetylation in chromatin structure and transcription. Nature.

[12] Lee K.K., Workman J.L. (2007). Histone acetyltransferase complexes: One size doesn’t fit all. Nat. Rev. Mol. Cell Biol..

[13] Jaliffa C., Ameqrane I., Dansault A., Leemput J., Vieira V., Lacassagne E., Provost A., Bigot K., Masson C., Menasche M. (2009). Sirt1 involvement in rd10 mouse retinal degeneration. Investig. Ophthalmol. Vis. Sci..

[14] Kubota S., Kurihara T., Mochimaru H., Satofuka S., Noda K., Ozawa Y., Oike Y., Ishida S., Tsubota K. (2009). Prevention of ocular inflammation in endotoxin-induced uveitis with resveratrol by inhibiting oxidative damage and nuclear factor-κB activation. Investig. Ophthalmol. Vis. Sci..

[15] Peng C.H., Chang Y.L., Kao C.L., Tseng L.M., Wu C.C., Chen Y.C., Tsai C.Y., Woung L.C., Liu J.H., Chiou S.H. (2010). SirT1—A sensor for monitoring self-renewal and aging process in retinal stem cells. Sensors.

[16] Anekonda T.S., Adamus G. (2008). Resveratrol prevents antibody-induced apoptotic death of retinal cells through upregulation of Sirt1 and Ku70. BMC Res. Notes.

[17] Chen D., Pacal M., Wenzel P., Knoepfler P.S., Leone G., Bremner R. (2009). Division and apoptosis of E2f-deficient retinal progenitors. Nature.

[18] Peng L., Yuan Z., Ling H., Fukasawa K., Robertson K., Olashaw N., Koomen J., Chen J., Lane W.S., Seto E. (2011). SIRT1 deacetylates the DNA methyltransferase 1 (DNMT1) protein and alters its activities. Mol. Cell. Biol..

[19] Silberman D.M., Ross K., Sande P.H., Kubota S., Ramaswamy S., Apte R.S., Mostoslavsky R. (2014). SIRT6 is required for normal retinal function. PLoS ONE.

[20] Zhang T., Cooper S., Brockdorff N. (2015). The interplay of histone modifications—Writers that read. EMBO Rep..

[21] Popova E.Y., Xu X., DeWan A.T., Salzberg A.C., Berg A., Hoh J., Zhang S.S., Barnstable C.J. (2012). Stage and gene specific signatures defined by histones H3K4me2 and H3K27me3 accompany mammalian retina maturation in vivo. PLoS ONE.

[22] Katoh K., Yamazaki R., Onishi A., Sanuki R., Furukawa T. (2012). G9a histone methyltransferase activity in retinal progenitors is essential for proper differentiation and survival of mouse retinal cells. J. Neurosci..

[23] Shi Y., Lan F., Matson C., Mulligan P., Whetstine J.R., Cole P.A., Casero R.A., Shi Y. (2004). Histone demethylation mediated by the nuclear amine oxidase homolog LSD1. Cell.

[24] Zheng S., Xiao L., Liu Y., Wang Y., Cheng L., Zhang J., Yan N., Chen D. (2018). DZNep inhibits H3K27me3 deposition and delays retinal degeneration in the rd1 mice. Cell. Death Dis..

[25] Aldiri I., Ajioka I., Xu B., Zhang J., Chen X., Benavente C., Finkelstein D., Johnson D., Akiyama J., Pennacchio L.A. (2015). Brg1 coordinates multiple processes during retinogenesis and is a tumor suppressor in retinoblastoma. Development.

[26] Lamba D.A., Hayes S., Karl M.O., Reh T. (2008). Baf60c is a component of the neural progenitor-specific BAF complex in developing retina. Dev. Dyn..

[27] Karali M., Banfi S. (2019). Non-coding RNAs in retinal development and function. Hum. Genet..

[28] Carrella S., Banfi S., Karali M. (2020). Sophisticated Gene Regulation for a Complex Physiological System: The Role of Non-coding RNAs in Photoreceptor Cells. Front. Cell. Dev. Biol..

[29] Friedman R.C., Farh K.K., Burge C.B., Bartel D.P. (2009). Most mammalian mRNAs are conserved targets of microRNAs. Genome Res..

[30] Lee Y., Kim M., Han J., Yeom K.H., Lee S., Baek S.H., Kim V.N. (2004). MicroRNA genes are transcribed by RNA polymerase II. EMBO J..

[31] Zeng Y., Yi R., Cullen B.R. (2005). Recognition and cleavage of primary microRNA precursors by the nuclear processing enzyme Drosha. EMBO J..

[32] Faller M., Toso D., Matsunaga M., Atanasov I., Senturia R., Chen Y., Zhou Z.H., Guo F. (2010). DGCR8 recognizes primary transcripts of microRNAs through highly cooperative binding and formation of higher-order structures. RNA.

[33] Schwarz D.S., Hutvagner G., Du T., Xu Z., Aronin N., Zamore P.D. (2003). Asymmetry in the assembly of the RNAi enzyme complex. Cell.

[34] Bartel D.P. (2009). MicroRNAs: Target recognition and regulatory functions. Cell.

[35] Fishman E.S., Han J.S., La Torre A. (2022). Oscillatory Behaviors of microRNA Networks: Emerging Roles in Retinal Development. Front. Cell. Dev. Biol..

[36] Xu S., Witmer P.D., Lumayag S., Kovacs B., Valle D. (2007). MicroRNA (miRNA) transcriptome of mouse retina and identification of a sensory organ-specific miRNA cluster. J. Biol. Chem..

[37] Horsford D.J., Nguyen M.T., Sellar G.C., Kothary R., Arnheiter H., McInnes R.R. (2005). Chx10 repression of Mitf is required for the maintenance of mammalian neuroretinal identity. Development.

[38] Zuzic M., Rojo Arias J.E., Wohl S.G., Busskamp V. (2019). Retinal miRNA Functions in Health and Disease. Genes.

[39] Busskamp V., Krol J., Nelidova D., Daum J., Szikra T., Tsuda B., Juttner J., Farrow K., Scherf B.G., Alvarez C.P. (2014). miRNAs 182 and 183 are necessary to maintain adult cone photoreceptor outer segments and visual function. Neuron.

[40] Pawlick J.S., Zuzic M., Pasquini G., Swiersy A., Busskamp V. (2020). MiRNA Regulatory Functions in Photoreceptors. Front. Cell. Dev. Biol..

[41] Krol J., Krol I., Alvarez C.P., Fiscella M., Hierlemann A., Roska B., Filipowicz W. (2015). A network comprising short and long noncoding RNAs and RNA helicase controls mouse retina architecture. Nat. Commun..

[42] Krol J., Busskamp V., Markiewicz I., Stadler M.B., Ribi S., Richter J., Duebel J., Bicker S., Fehling H.J., Schubeler D. (2010). Characterizing light-regulated retinal microRNAs reveals rapid turnover as a common property of neuronal microRNAs. Cell.

[43] Bringmann A., Pannicke T., Grosche J., Francke M., Wiedemann P., Skatchkov S.N., Osborne N.N., Reichenbach A. (2006). Muller cells in the healthy and diseased retina. Prog. Retin. Eye Res..

[44] La Torre A., Georgi S., Reh T.A. (2013). Conserved microRNA pathway regulates developmental timing of retinal neurogenesis. Proc. Natl. Acad. Sci. USA.

[45] Young T.L., Matsuda T., Cepko C.L. (2005). The noncoding RNA taurine upregulated gene 1 is required for differentiation of the murine retina. Curr. Biol..

[46] Alfano G., Vitiello C., Caccioppoli C., Caramico T., Carola A., Szego M.J., McInnes R.R., Auricchio A., Banfi S. (2005). Natural antisense transcripts associated with genes involved in eye development. Hum. Mol. Genet..

[47] Meola N., Pizzo M., Alfano G., Surace E.M., Banfi S. (2012). The long noncoding RNA Vax2os1 controls the cell cycle progression of photoreceptor progenitors in the mouse retina. RNA.

[48] Wang Y., Wang X., Wang Y.X., Ma Y., Di Y. (2020). Effect and mechanism of the long noncoding RNA MALAT1 on retinal neovascularization in retinopathy of prematurity. Life Sci..

[49] Zhang Y.L., Hu H.Y., You Z.P., Li B.Y., Shi K. (2020). Targeting long non-coding RNA MALAT1 alleviates retinal neurodegeneration in diabetic mice. Int. J. Ophthalmol..

[50] Chen X.J., Zhang Z.C., Wang X.Y., Zhao H.Q., Li M.L., Ma Y., Ji Y.Y., Zhang C.J., Wu K.C., Xiang L. (2020). The Circular RNome of Developmental Retina in Mice. Mol. Ther. Nucleic Acids.

[51] Solovei I., Kreysing M., Lanctot C., Kosem S., Peichl L., Cremer T., Guck J., Joffe B. (2009). Nuclear architecture of rod photoreceptor cells adapts to vision in mammalian evolution. Cell.

[52] Rembold M., Loosli F., Adams R.J., Wittbrodt J. (2006). Individual cell migration serves as the driving force for optic vesicle evagination. Science.

[53] Cvekl A., Mitton K.P. (2010). Epigenetic regulatory mechanisms in vertebrate eye development and disease. Heredity.

[54] Rapaport D.H., Wong L.L., Wood E.D., Yasumura D., LaVail M.M. (2004). Timing and topography of cell genesis in the rat retina. J. Comp. Neurol..

[55] Zhou G., Strom R.C., Giguere V., Williams R.W. (2001). Modulation of retinal cell populations and eye size in retinoic acid receptor knockout mice. Mol. Vis..

[56] Cvekl A., Wang W.L. (2009). Retinoic acid signaling in mammalian eye development. Exp. Eye Res..

[57] Swaroop A., Kim D., Forrest D. (2010). Transcriptional regulation of photoreceptor development and homeostasis in the mammalian retina. Nat. Rev. Neurosci..

[58] Chen B., Cepko C.L. (2007). Requirement of histone deacetylase activity for the expression of critical photoreceptor genes. BMC Dev. Biol..

[59] Margueron R., Reinberg D. (2011). The Polycomb complex PRC2 and its mark in life. Nature.

[60] Marquardt T., Ashery-Padan R., Andrejewski N., Scardigli R., Guillemot F., Gruss P. (2001). Pax6 is required for the multipotent state of retinal progenitor cells. Cell.

[61] Mo A., Luo C., Davis F.P., Mukamel E.A., Henry G.L., Nery J.R., Urich M.A., Picard S., Lister R., Eddy S.R. (2016). Epigenomic landscapes of retinal rods and cones. Elife.

[62] Rhee K.D., Yu J., Zhao C.Y., Fan G., Yang X.J. (2012). Dnmt1-dependent DNA methylation is essential for photoreceptor terminal differentiation and retinal neuron survival. Cell Death Dis..

[63] Rai K., Jafri I.F., Chidester S., James S.R., Karpf A.R., Cairns B.R., Jones D.A. (2010). Dnmt3 and G9a cooperate for tissue-specific development in zebrafish. J. Biol. Chem..

[64] Yang Y., Wolf L.V., Cvekl A. (2007). Distinct embryonic expression and localization of CBP and p300 histone acetyltransferases at the mouse alphaA-crystallin locus in lens. J. Mol. Biol..

[65] Quintero H., Lamas M. (2018). microRNA expression in the neural retina: Focus on Muller glia. J. Neurosci. Res..

[66] Bueno M.J., Malumbres M. (2011). MicroRNAs and the cell cycle. Biochim. Biophys. Acta.

[67] Xia X., Ahmad I. (2016). let-7 microRNA regulates neurogliogenesis in the mammalian retina through Hmga2. Dev. Biol..

[68] Peskova L., Jurcikova D., Vanova T., Krivanek J., Capandova M., Sramkova Z., Sebestikova J., Kolouskova M., Kotasova H., Streit L. (2020). miR-183/96/182 cluster is an important morphogenetic factor targeting PAX6 expression in differentiating human retinal organoids. Stem Cells.

[69] Sanuki R., Onishi A., Koike C., Muramatsu R., Watanabe S., Muranishi Y., Irie S., Uneo S., Koyasu T., Matsui R. (2011). miR-124a is required for hippocampal axogenesis and retinal cone survival through Lhx2 suppression. Nat. Neurosci..

[70] Bonev B., Stanley P., Papalopulu N. (2012). MicroRNA-9 Modulates Hes1 ultradian oscillations by forming a double-negative feedback loop. Cell Rep..

[71] Suuronen T., Nuutinen T., Ryhanen T., Kaarniranta K., Salminen A. (2007). Epigenetic regulation of clusterin/apolipoprotein J expression in retinal pigment epithelial cells. Biochem. Biophys. Res. Commun..

[72] Hunter A., Spechler P.A., Cwanger A., Song Y., Zhang Z., Ying G.S., Hunter A.K., Dezoeten E., Dunaief J.L. (2012). DNA methylation is associated with altered gene expression in AMD. Investig. Ophthalmol. Vis. Sci..

[73] Wei L., Liu B., Tuo J., Shen D., Chen P., Li Z., Liu X., Ni J., Dagur P., Sen H.N. (2012). Hypomethylation of the IL17RC promoter associates with age-related macular degeneration. Cell Rep..

[74] Porter L.F., Saptarshi N., Fang Y., Rathi S., den Hollander A.I., de Jong E.K., Clark S.J., Bishop P.N., Olsen T.W., Liloglou T. (2019). Whole-genome methylation profiling of the retinal pigment epithelium of individuals with age-related macular degeneration reveals differential methylation of the SKI, GTF2H4, and TNXB genes. Clin. Epigenet..

[75] Chu-Tan J.A., Rutar M., Saxena K., Aggio-Bruce R., Essex R.W., Valter K., Jiao H., Fernando N., Wooff Y., Madigan M.C. (2018). MicroRNA-124 Dysregulation is Associated With Retinal Inflammation and Photoreceptor Death in the Degenerating Retina. Investig. Ophthalmol. Vis. Sci..

[76] Olivares A.M., Jelcick A.S., Reinecke J., Leehy B., Haider A., Morrison M.A., Cheng L., Chen D.F., DeAngelis M.M., Haider N.B. (2017). Multimodal Regulation Orchestrates Normal and Complex Disease States in the Retina. Sci. Rep..

[77] Yu X., Luo Y., Chen G., Liu H., Tian N., Zen X., Huang Y. (2021). Long non-coding RNA PWRN2 regulates cytotoxicity in an in vitro model of age-related macular degeneration. Biochem. Biophys. Res. Commun..

[78] Chen X., Sun R., Yang D., Jiang C., Liu Q. (2020). LINC00167 Regulates RPE Differentiation by Targeting the miR-203a-3p/SOCS3 Axis. Mol. Ther. Nucleic Acids.

[79] Zhou R.M., Shi L.J., Shan K., Sun Y.N., Wang S.S., Zhang S.J., Li X.M., Jiang Q., Yan B., Zhao C. (2020). Circular RNA-ZBTB44 regulates the development of choroidal neovascularization. Theranostics.

[80] Boukhtouche F., Mariani J., Tedgui A. (2004). The “CholesteROR” protective pathway in the vascular system. Arterioscler. Thromb. Vasc. Biol..

[81] Lau P., Fitzsimmons R.L., Raichur S., Wang S.C., Lechtken A., Muscat G.E. (2008). The orphan nuclear receptor, RORalpha, regulates gene expression that controls lipid metabolism: Staggerer (SG/SG) mice are resistant to diet-induced obesity. J. Biol. Chem..

[82] Han S., Li Z., Han F., Jia Y., Qi L., Wu G., Cai W., Xu Y., Li C., Zhang W. (2019). ROR alpha protects against LPS-induced inflammation by down-regulating SIRT1/NF-κ B pathway. Arch. Biochem. Biophys..

[83] Delerive P., Monte D., Dubois G., Trottein F., Fruchart-Najib J., Mariani J., Fruchart J.C., Staels B. (2001). The orphan nuclear receptor ROR alpha is a negative regulator of the inflammatory response. EMBO Rep..

[84] Lee J.M., Kim H., Baek S.H. (2021). Unraveling the physiological roles of retinoic acid receptor-related orphan receptor alpha. Exp. Mol. Med..

[85] Silveira A.C., Morrison M.A., Ji F., Xu H., Reinecke J.B., Adams S.M., Arneberg T.M., Janssian M., Lee J.E., Yuan Y. (2010). Convergence of linkage, gene expression and association data demonstrates the influence of the RAR-related orphan receptor alpha (RORA) gene on neovascular AMD: A systems biology based approach. Vision Res..

[86] Lukiw W.J., Surjyadipta B., Dua P., Alexandrov P.N. (2012). Common micro RNAs (miRNAs) target complement factor H (CFH) regulation in Alzheimer’s disease (AD) and in age-related macular degeneration (AMD). Int. J. Biochem. Mol. Biol..

[87] Hao Y., Zhou Q., Ma J., Zhao Y., Wang S. (2016). miR-146a is upregulated during retinal pigment epithelium (RPE)/choroid aging in mice and represses IL-6 and VEGF-A expression in RPE cells. J. Clin. Exp. Ophthalmol..

[88] Wahlin K.J., Enke R.A., Fuller J.A., Kalesnykas G., Zack D.J., Merbs S.L. (2013). Epigenetics and cell death: DNA hypermethylation in programmed retinal cell death. PLoS ONE.

[89] Farinelli P., Perera A., Arango-Gonzalez B., Trifunovic D., Wagner M., Carell T., Biel M., Zrenner E., Michalakis S., Paquet-Durand F. (2014). DNA methylation and differential gene regulation in photoreceptor cell death. Cell Death Dis..

[90] Sancho-Pelluz J., Alavi M.V., Sahaboglu A., Kustermann S., Farinelli P., Azadi S., van Veen T., Romero F.J., Paquet-Durand F., Ekstrom P. (2010). Excessive HDAC activation is critical for neurodegeneration in the rd1 mouse. Cell Death Dis..

[91] Paquet-Durand F., Silva J., Talukdar T., Johnson L.E., Azadi S., van Veen T., Ueffing M., Hauck S.M., Ekstrom P.A. (2007). Excessive activation of poly(ADP-ribose) polymerase contributes to inherited photoreceptor degeneration in the retinal degeneration 1 mouse. J. Neurosci..

[92] Samardzija M., Corna A., Gomez-Sintes R., Jarboui M.A., Armento A., Roger J.E., Petridou E., Haq W., Paquet-Durand F., Zrenner E. (2021). HDAC inhibition ameliorates cone survival in retinitis pigmentosa mice. Cell Death Differ..

[93] Chen B., Cepko C.L. (2009). HDAC4 regulates neuronal survival in normal and diseased retinas. Science.

[94] Duraisamy A.J., Mishra M., Kowluru A., Kowluru R.A. (2018). Epigenetics and Regulation of Oxidative Stress in Diabetic Retinopathy. Investig. Ophthalmol. Vis. Sci..

[95] Li S., Datta S., Brabbit E., Love Z., Woytowicz V., Flattery K., Capri J., Yao K., Wu S., Imboden M. (2021). Nr2e3 is a genetic modifier that rescues retinal degeneration and promotes homeostasis in multiple models of retinitis pigmentosa. Gene Ther..

[96] Gire A.I., Sullivan L.S., Bowne S.J., Birch D.G., Hughbanks-Wheaton D., Heckenlively J.R., Daiger S.P. (2007). The Gly56Arg mutation in NR2E3 accounts for 1–2% of autosomal dominant retinitis pigmentosa. Mol. Vis..

[97] Loscher C.J., Hokamp K., Wilson J.H., Li T., Humphries P., Farrar G.J., Palfi A. (2008). A common microRNA signature in mouse models of retinal degeneration. Exp. Eye Res..

[98] Leyk J., Daly C., Janssen-Bienhold U., Kennedy B.N., Richter-Landsberg C. (2017). HDAC6 inhibition by tubastatin A is protective against oxidative stress in a photoreceptor cell line and restores visual function in a zebrafish model of inherited blindness. Cell Death Dis..

[99] Zhang H., Dai X., Qi Y., He Y., Du W., Pang J.J. (2015). Histone Deacetylases Inhibitors in the Treatment of Retinal Degenerative Diseases: Overview and Perspectives. J. Ophthalmol..

[100] Ong T., Pennesi M.E., Birch D.G., Lam B.L., Tsang S.H. (2019). Adeno-Associated Viral Gene Therapy for Inherited Retinal Disease. Pharm. Res..

[101] Rajanala K., Dotiwala F., Upadhyay A. (2023). Geographic atrophy: Pathophysiology and current therapeutic strategies. Front. Ophthalmol..

[102] Akula M., McNamee S., Chan N.P.M., DeAngelis M.M., Haider N.B. (2023). RORA Modifier Gene Therapy Rescues Retinal Degeneration in a Juvenile AMD Mouse Model of Stargardt Disease. Investig. Ophthalmol. Vis. Sci..

[103] Karali M., Guadagnino I., Marrocco E., De Cegli R., Carissimo A., Pizzo M., Casarosa S., Conte I., Surace E.M., Banfi S. (2020). AAV-miR-204 Protects from Retinal Degeneration by Attenuation of Microglia Activation and Photoreceptor Cell Death. Mol. Ther. Nucleic Acids.

